# The Necessity of Interoperability to Uncover the Full Potential of Digital Health Devices

**DOI:** 10.2196/49301

**Published:** 2023-12-22

**Authors:** Julian D Schwab, Silke D Werle, Rolf Hühne, Hannah Spohn, Udo X Kaisers, Hans A Kestler

**Affiliations:** 1 Ulm University Ulm Germany; 2 University Hospital Ulm Ulm Germany

**Keywords:** semantic terminology, semantic, terminology, terminologies, data linkage, interoperability, data exchange, SNOMED CT, LOINC, eHealth, patient-reported outcome questionnaires, requirement for standards, standard, standards, PRO, PROM, patient reported

## Abstract

Personalized health care can be optimized by including patient-reported outcomes. Standardized and disease-specific questionnaires have been developed and are routinely used. These patient-reported outcome questionnaires can be simple paper forms given to the patient to fill out with a pen or embedded in digital devices. Regardless of the format used, they provide a snapshot of the patient’s feelings and indicate when therapies need to be adjusted. The advantage of digitizing these questionnaires is that they can be automatically analyzed, and patients can be monitored independently of doctor visits. Although the questions of most clinical patient-reported outcome questionnaires follow defined standards and are evaluated by clinical trials, these standards do not exist for data processing. Interoperable data formats and structures would benefit multilingual and cross-study data exchange. Linking questionnaires to standardized terminologies such as the Systematized Nomenclature of Medicine Clinical Terms (SNOMED CT) and Logical Observation Identifiers, Names, and Codes (LOINC) would improve this interoperability. However, linking clinically validated patient-reported outcome questionnaires to clinical terms available in SNOMED CT or LOINC is not as straightforward as it sounds. Here, we report our approach to link patient-reported outcomes from health applications to SNOMED CT or LOINC codes. We highlight current difficulties in this process and outline ways to minimize them.

## Introduction

The recording of symptoms and laboratory tests is of paramount importance in clinical practice. In addition to objective, measurable parameters, the patient-reported symptoms and their quality of life have come into focus in modern medicine [1,2]. In this context, patient-reported outcome (PRO) questionnaires have become a standard tool to capture patients’ perspectives on their health status. Depending on the underlying disease, PROs cover topics such as quality of life [1], adverse events [3,4], or stress [5], among others. Although in the past, PROs were typically reported using paper-and-pencil methods, electronic PROs (ePROs) are increasingly used and preferred by patients [6]. Because physician and patient perceptions of symptoms can be discrepant [7], PROs are essential for identifying conditions requiring a therapy adjustment [3]. In addition, ePROs can automate their analysis, and they can be used as an outpatient triage tool to identify those in a cohort who need therapy adjustment or closer support [8]. PRO questionnaires can be constructed for different topics and can be more general or tailored to specific diseases [9]. In addition, questions can have different recall time resolutions (eg, today or last week) or differ in their detailed description of symptoms [3]. Thus, the questions and the corresponding answers can be very specific. As a result, minor adjustments in wording can affect the final result [9]. The same is valid for alternative translations. To address these issues, the European Organization for Research and Treatment of Cancer (EORTC), among others, has developed standardized quality-of-life PRO questionnaires [1]. These questionnaires were initially developed in 1986 and have been refined, translated into several languages, and validated [10,11]. Moreover, the consortium provides a detailed analysis plan to enable comparable results between clinical trials regardless of the geographical region or linguistic and cultural population [10].

**Figure 1:**
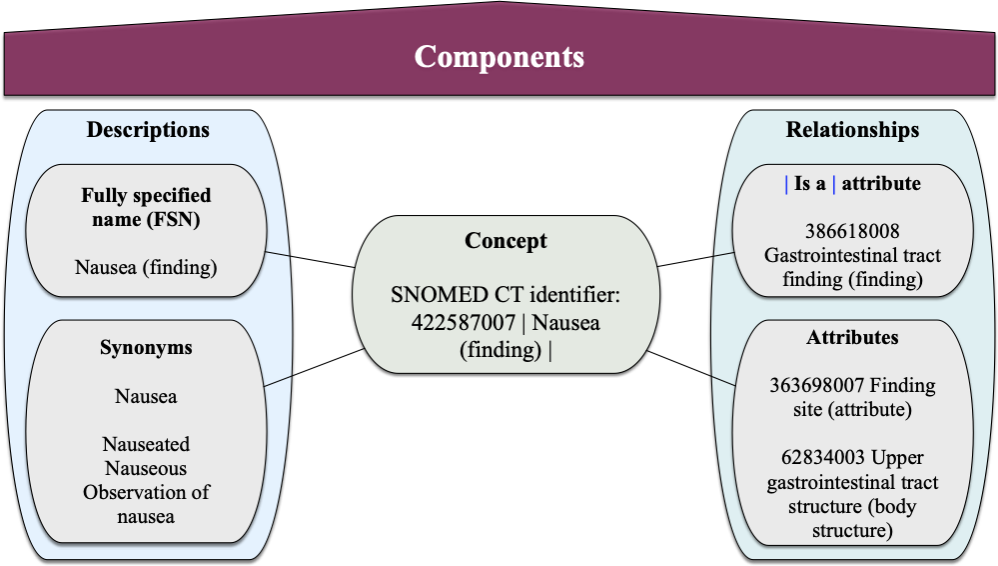
Exemplary composition of SNOMED CT components for nausea. SNOMED CT content consists of concepts, descriptions, and relationships. Each concept is associated with a set of textual descriptions. The descriptions can be grouped into an FSN, which is unique, and several accepted synonyms, allowing users to apply their preferred terms. In addition to the preferred FSN, a synonym can be selected by a language refset. Relationships link concepts within hierarchies. The |Is a| relationship connects concepts within the same hierarchy, whereas the attribute relationship connects relationships in different hierarchies such as finding site, procedure site, or method. SNOMED CT: Systematized Nomenclature of Medicine Clinical Terms.

**Figure 2:**
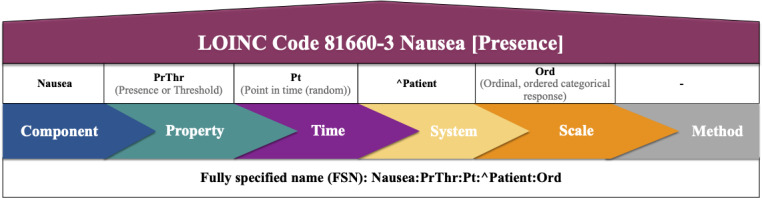
Exemplary composition of a LOINC term for nausea. A LOINC term is defined as the combination of the LOINC code, a unique identifier, and the FSN, which consists of 5-6 parts, including the component or analyte, the observed property, the time of the measurement, the type of system, the scale of the measurement, and sometimes also the method used. The FSN parts are listed sequentially, separated by “:”. “Nausea [Presence]” is one of the additional names, a long common name, which must be unique. LOINC: Logical Observation Identifiers, Names, and Codes.

Like the underlying questionnaires, the outgoing ePRO data should be accurate, standardized, and interoperable to enable sharing, reuse, and international data comparison. Unfortunately, what seems obvious at first glance is far from reality [12]. The Fast Healthcare Interoperability Resources (FHIR) specification, developed by Health Level Seven International, is the most widely accepted standard for communicating health care data [12]. It aims to provide a comprehensive framework and related standards for exchanging, integrating, sharing, and retrieving electronic health information [13]. The FHIR elements required for PRO questionnaires are Questionnaire and QuestionnaireResponse. A Questionnaire defines a structured set of questions to guide the collection of answers. In addition to the questions, it also defines the answer types and possible answers. It provides detailed control over order, presentation, phraseology, and grouping for consistent data collection. Each Questionnaire requires a QuestionnaireResponse as its counterpart for retrieving and organizing the answers. The QuestionnaireResponse provides a structured set of answers for a specific Questionnaire and must match the definitions of the Questionnaire. Terminologies are integrated as CodeSystems by providing a base URL and the term code. This combines the syntactic interoperability (structure and data format) enabled by standards such as FHIR with the semantic interoperability enabled by health terminologies. The Systematized Nomenclature of Medical Clinical Terms (SNOMED CT) is currently the most appropriate and comprehensive clinical health terminology with natural language properties [14,15]. It includes terminologies for medical concepts, descriptions, and relationships, forming a unique component with a specific identifier [15,16] (Figure 1). Another common medical terminology is the Logical Observation Identifiers Names and Codes (LOINC) [12,14]. A LOINC term is defined as the combination of the LOINC code, a unique identifier, and the fully specified name (FSN), which consists of 5 to 6 parts, including the component or analyte, the observed property, the time of the measurement, the type of system, and the scale of the measurement. Where relevant, the measurement method is included as part 6 [17] (Figure 2). Although SNOMED CT seems appropriate as a reference terminology for multilingual semantic interoperability in health care [18], LOINC has the advantage of also providing “display text” that can be used to link questions (“SURVEY_QUEST_TEXT”) [17]. For these reasons, it may be appropriate to supplement SNOMED CT terminology with LOINC, and several approaches for their mapping have been proposed [19,20].

To improve the international reuse and comparison of PROs in clinical trials, we tested the mapping of standardized and validated questionnaires with their corresponding queries to the SNOMED CT and LOINC terminologies. In the following sections, we introduce these PROs, report on our experience mapping them to SNOMED CT and LOINC, highlight current difficulties, and outline some suggestions for minimizing them.

## Patient-Reported Outcome Questionnaires

### Content of Patient-Reported Outcome Questionnaires

Various questionnaires focus either on the quality of life and its impact on the underlying disease or on disease- or treatment-related adverse events. Recent approaches have identified conceptual differences between these PRO measures (PROMs) [21]. On the basis of these findings, we have limited our approach to a few clinically relevant and widely accepted questionnaires that differ in their representation of the domains assessed. Thus, our content analysis included the following PROMs, which can be accessed at

- EORTC Quality of Life of Cancer Patients Questionnaire (QLQ-C30) [22]
- EORTC Quality of Life of Breast Cancer Patients Questionnaire (QLQ-BR23) [22]
- Patient-Reported Outcomes Measurement Information System (PROMIS-29) [23]
- Common Terminology Criteria for Adverse Events (CTCAE) [24].

In the following, we describe the analyzed PROMs in more detail.

### EORTC QLQ-C30

The EORTC QLQ-C30 is one of the core validated questionnaires of the EORTC Quality of Life Group. It has been optimized in several revisions since its initial release in 1987 and assesses general aspects and symptoms of patients with cancer based on 30 core items [1]. As there is a wide range of tumor diseases, the questionnaire is not considered to be too disease specific. Five questions of the QLQ-C30 do not consider a recall time interval, whereas the remaining questions refer to the recall time interval *last week.* Two different value scales are used for the answers. Most of the questions can be answered with the text *not at all*, *a little*, *quite a bit*, and *very much*, combined with the numerical values 1 to 4. The others have only text for the first and last values (*very poor* and *excellent*), and the numerical values range from 1 to 7. To score the QLQ-C30, the questions are divided into groups to assess functional scales, symptom-related scales, and global health status, using the Likert method of summing scales [10]. The QLQ-C30 is currently available in version 3.0 and is recommended for use in any new study [10]. The QLQ-C30 core questionnaire can be supplemented by modules containing questions about specific tumor sites, symptoms, or treatments [10,25]. All EORTC questionnaires are available in several languages, but the initial version is in English [10,26]. Special care has been taken in their development to ensure that they are universally understandable, regardless of the level of education or cultural background. To ensure these quality standards, each questionnaire undergoes several rounds of revision, including testing with patients from several countries [26]. The current versions of all EORTC questionnaires and their development status can be requested at the website in [22].

### EORTC QLQ-BR23

The EORTC QLQ-BR23 questionnaire is the validated add-on module to the QLQ-C30 core questionnaire for patients with breast cancer. It is recommended for general use, but its updated version (QLQ-BR45) has already completed phase IV testing [10], and a new version will soon be available for newly initiated studies. The QLQ-BR23 questionnaire consists of 23 items that address dealing with symptoms, treatment adverse events, body image, sexual functioning, and future outlook [10]. Similar to the QLQ-C30, the items of the QLQ-BR23 can be grouped for scoring based on the available scoring scheme [10]. This time, however, all questions can be answered with the text *not at all*, *a little*, *quite a bit,* and *very much* combined with the numerical values 1 to 4, and the questions cover a recall time interval of either the last week or the last 4 weeks.

### PROMIS-29

The PROMIS is an item bank for constructing patient-reported questionnaires in the context of chronic diseases and conditions. It covers the domains of physical, mental, and social health, which are further subdivided into more precise symptom item banks such as physical function (124 items), pain behavior (39 items), or fatigue (95 items), among others, tailored to a broad population of chronic diseases. This allows comparisons between diseases. The system enables exchanging items or minimizing the number of items within a questionnaire without compromising the reliability [27].

Because of the many possible PROMIS questionnaires, we limited our approach to the PROMIS-29 questionnaire available at the website in [23]. It contains 29 questions derived from the 7 PROMIS categories of physical function, anxiety, depression, fatigue, sleep disturbance, ability to participate in social roles and activities, and pain interference, each measured by 4 questions. It also includes a numeric pain intensity scale ranging from 0 (no pain) to 10 (worst pain imaginable). In addition to the pain intensity scale, all other items have a 5-point response scale (eg, 1=never, 2=rarely, 3=sometimes, 4=often, and 5=always). Except for the questions on physical function and ability to participate in social roles and activities, where the time interval is not specified, the requested recall period is the past 7 days.

### CTCAE

The CTCAE is the classic scoring system physicians use to classify side effects during cancer treatment. It is used to grade patient-reported symptoms and those observed by clinicians or from laboratory tests [7,28]. Unlike the EORTC and PROMIS questionnaires, the CTCAE questions were not originally designed in a pen-and-paper format to be given to patients. Therefore, clinicians do not rate each question. It is a descriptive terminology for adverse events coupled with a descriptive grading of the event that occurred [24]. The CTCAE presents scores from 1 to 5 with the associated grading (*mild*, *moderate*, *severe*, *life-threatening*, or *dead*). Each grade has a unique textual clinical description to help to assess the correct grading. Because not all adverse events can be classified into 5 classes, some described adverse events have fewer than 5 grades [24]. The current CTCAE scoring (version 5.0) and previous versions are available at the website in [29]. We have limited our approach to the current CTCAE version 5.0. However, a CTCAE version 6.0 is already in preparation. Following the idea of the CTCAE, the National Cancer Institute (NCI) has developed a Patient-Reported Outcomes Version of the CTCAE (PRO-CTCAE) scoring system. It contains 78 adverse events from the classic CTCAE, which can be queried based on 124 individual questions [2,30]. The PRO-CTCAE scoring system records adverse events according to attributes such as severity (*none*, *mild*, *moderate*, *severe*, *very severe*), frequency (*never*, *rarely*, *occasionally*, *frequently*, *almost constantly*), or interference (*not at all*, *a little bit*, *somewhat*, *quite a bit*, *very much*) and has a general recall time of the last 7 days [30]. Thus, it lacks the detailed textual descriptions of the symptoms that occurred. In contrast, the health application NEMO (German, Nebenwirkungs-Management Onkologie) tried to combine PRO questions with the classic CTCAE scoring descriptions for daily use [3].

## Workflow—Finding SNOMED CT and LOINC Codes for PRO Questionnaires

### Limitations

SNOMED CT and LOINC are both optimized for health care and laboratory terminologies. Thus, several approaches have been proposed to map SNOMED CT and LOINC codes [19,20] due to their close knowledge representation formalisms (see Figures 1 and 2). However, even applying this mapping to data for which both terminologies are specialized resulted in incomplete results and required significant human effort to complete the task [19,20].

**Figure 3:**
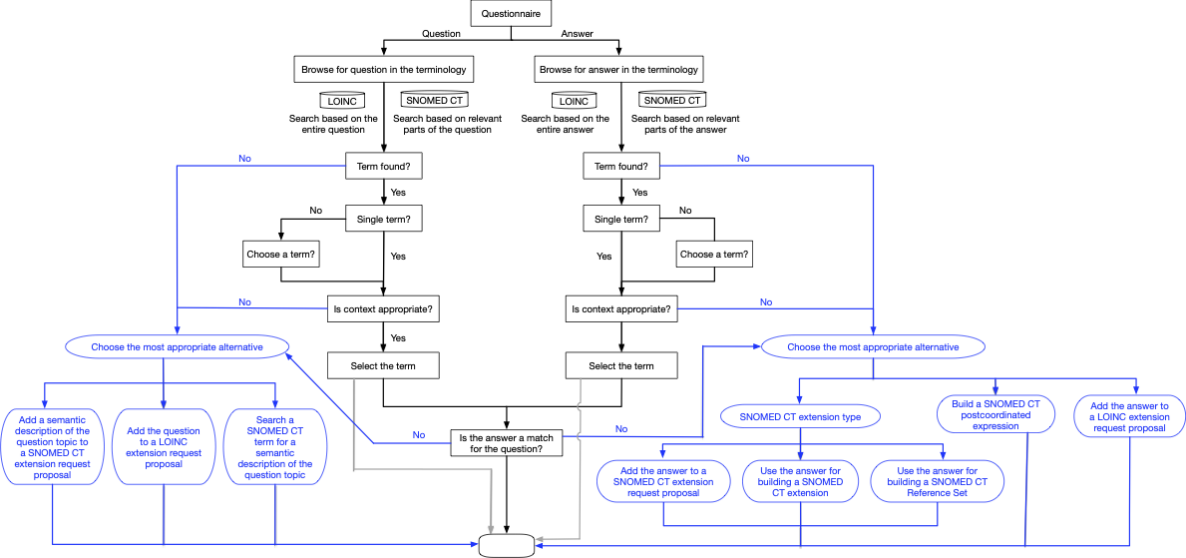
Workflow. Depicted is our workflow to match patient-reported outcome questionnaires with the standardized terminologies LOINC and SNOMET CT to enable semantic interoperability. The workflow marked in black depicts the procedure to analyze the current status of terms included in both terminologies. If a term still needs to be included, we suggest the blue workflow to extend the terminologies. The empty box indicates the end of the procedure. LOINC: Logical Observation Identifiers, Names, and Codes; SNOMED CT: Systematized Nomenclature of Medicine Clinical Terms.

Therefore, we also manually mapped the questionnaires with their associated questions and response options to assign them SNOMED CT and LOINC codes. The questionnaires analyzed in our study were obtained from [22-24]. The workflow used is shown in Figure 3 and is described in more detail below. The results of the manual mapping can be found in Tables S1-S4 in Multimedia Appendix 1.

We did not consider the relationship between questions and responses in our analyses of term availability. However, we want to inform the reader that the context between questions and possible responses must also be considered and marked in our workflow for actual use cases.

### LOINC

After free registration at the website in [31], keywords from our PRO questions and possible answers were queried. If only 1 term was found, it was evaluated if it was a “perfect match.” If multiple terms were found, the most appropriate term was selected and considered a perfect match. If this evaluation resulted in a perfect match, the term was chosen as the terminology term. However, if the match was negative or no term was found, the item was treated as “not found.” For example, for the question *Have you felt nauseated?* there was no match, and a search for the term *nausea* alone returned 12 results, which are listed as follows:

- *67232-9 | How often did you have nausea in the past 7 days [PhenX]*
- *64713-1 | In which months of the pregnancy did you have frequent nausea or vomiting [PhnX]*
- *81660-3 | Nausea [Presence]*
- *70406-4 | I have nausea in the past 7 days [FACIT]*
- *77711-0 | Patient has anorexia, nausea or vomiting in the past week [UPDRS]*
- *42848-2 | Nausea [CCC]*
- *77510-6 | Can pronounce nausea [AmNART]*
- *28391-1 | Nausea [HIV-SSC]*
- *69711-0 | Did you have nausea or an upset stomach, or the feeling that you were going to have diarrhea [Reported.PHQ]*
- *69682-3 | Bothered by nausea, gas, or indigestion in last 4 weeks [Reported.PHQ]*
- *67231-1 | How often did you have pain in the center of the upper stomach in the past 7 days [PhenX]*
- *96839-6 | Nauseous or had stomach problems when I thought about or was exposed to information about the coronavirus in past 2 weeks*

All of these are not a perfect match to the original question. Therefore, we considered the term not an ideal match.

### SNOMED CT

To find SNOMED CT components and the corresponding identifier, we used the SNOMED CT Browser [32] and entered keywords from our PRO questions and possible answers into the search field.

Because full text cannot be entered into SNOMED CT, we searched for relevant parts of the question or answer. For example, for the question *Have you felt nauseated?* the search term *nauseated* or *nausea* returned the following result, which we interpret as a perfect match:

- *422587007 | Nausea (finding) |*

Here, we were interested in a general proof of principle of whether PRO questionnaires can be mapped to semantic terminologies. Therefore, we stopped our analysis after querying the PRO questionnaire terms. However, if the goal is to extend the terminologies, we recommend the following alternatives (marked in blue in Figure 3), which we discuss in more detail below.

In the case of LOINC, the missing term can either be requested to be added by an extension request proposal, or a semantic description of the term can be searched in the SNOMED CT terminology. The specificity of the term description can be further increased by building a postcoordinated expression.

In the following sections, we report on the challenges of term mapping to semantic terminologies.

## Challenges in Terminology Binding

Our attempts to complete the exemplary questionnaires’ terminology binding revealed the following challenges.

### Language

Although the PRO questionnaires are translated into different languages while preserving the original meaning [11], the terminology databases SNOMED CT and LOINC are only available for a limited number of languages [18]. This is counterproductive to the goal of digitization and medical terminologies to improve interoperability, especially if automatic mapping is the ultimate goal. In addition to the international edition of SNOMED CT, there are currently 18 other country-specific editions that cover, at least in part, 10 additional languages: Danish, Dutch, Estonian, Finnish, French, German, Māori, Norwegian, Spanish, and Swedish [32]. However, several European languages are not yet included [18]. On the positive side, at least the same identifiers are used for different languages. However, this should be the case to ensure interoperability. LOINC currently has 20 linguistic variants covering 14 additional languages: Chinese, Dutch, Estonian, French, German, Greek, Italian, Korean, Polish, Portuguese, Russian, Spanish, Turkish, and Ukrainian [33]. Because of the various language alternatives, we limited our mapping to the English versions of the PRO questionnaires and terminology databases, as has been done by others [34]. The language barrier could be reduced by putting more effort into translating the terminologies into more languages. As mentioned above, FHIR, a standard for health data exchange, enables syntactic interoperability [13]. To ensure a correct transition to semantic interoperability, mapping validators, such as the open-source FHIR Validator framework [35], check whether the exported textual questionnaire and the specified identifier match. If the language of the PRO questionnaire and, thus, the survey text from the FHIR export differs from the language of the associated terminology, the program generates a warning. However, the fact that it only generates a warning may allow existing tools to continue to be used.

### Concept Availability and Term Selection

The SNOMED CT and LOINC terminologies are comprehensive, but questionnaire questions are often use-case-specific. We limited our approach to searching for general term matches in the terminologies to provide a proof of concept. However, we recognize and want to emphasize that terminology mapping of PRO questionnaires is more than just finding a match for applied use case scenarios. In addition to the term itself, the context must also match. For example, in SNOMED CT, the term *nausea* is a finding that expects a *yes* or *no* response. In contrast, in LOINC, the code *81660-3 | Nausea [Presence]* expects responses such as follows:

- *LA137-2 | None*
- *LA6752-5 | Mild*
- *LA6751-7 | Moderate*
- *LA6750-9 | Severe*
- *LA9041-0 | Resolved*

Therefore, the question and the response must be addressed in the correct context for a proper representation using encoding systems. This also includes the evaluation of multiple isosemantic representations.

In addition to the context, precise wording is paramount for PROs [25]. Mapping the PROMIS-29 questionnaire to LOINC version 2.71 was relatively straightforward because it has its own LOINC profile (*62337-1 | PROMIS item bank - 29 profile*), including codes for questions and response scores. However, caution is still required. Although the LOINC code for the PROMIS-29 panel has basic attributes, including first released and last updated, these version numbers do not match the version number of the underlying questionnaire. The PROMIS-29 questionnaire is available in versions 1.0, 2.0, and 2.1 [36], with changes to the *abilities to participate in social roles and activities* items in versions 2.0 and later. The available LOINC panel, instead, contains only the 1.0 version items. However, the 4 questions that have changed between these versions can also be found in another LOINC panel (*76731-9 | PROMIS short form - ability to participate in social roles and activities 8a - version 2.0*).

In contrast to LOINC, none of the complete PROMIS-29 statements could be mapped to SNOMED CT identifiers. Sometimes, the requested symptom finding could be matched, but without having the full statement (eg, SNOMED CT code: *307077003 | feeling hopeless (finding)|* for the PROMIS-29 statement *In the past seven days I felt hopeless*). However, not all descriptive symptoms (eg, *In the past seven days I felt worthless*) could be found in SNOMED CT. The best SNOMED CT match for the PROMIS-29 response options was obtained with the following:

- The parent concept: *1157335009 | Numeric grade on a scale of 1 to 5 (qualifier)|*
- Its child concept: *1157337001 | Grade 1 on a scale of 1 to 5 (qualifier value)|*
- Up to its child concept: *1157341002 | Grade 5 on a scale of 1 to 5 (qualifier value)|*
- The parent concept: *1157336005 | Numeric grade on a scale of 0 to 10 (qualifier value)|*

Another difficulty is that not all answer options in the PROMIS-29 questionnaire are associated with an ascending scale from 1 to 5. Nevertheless, this scale is sometimes reversed, even though the same text has been assigned. This demonstrates the disadvantage of using only numerical scales alone as terminological concepts for PRO comparison. Instead, it is essential to couple the numerical scales with a semantic representation of their meaning.

For the EORTC QLQ-C30 and QLQ-BR23 questionnaires, finding LOINC terms that semantically matched the questions was difficult. If at all, we could only find phrases formulated as statements that appeared in questions on the EORTC questionnaires, were taken from other questionnaires, and approximated the flow of words. For example, the QLQ-C30 question *Did you need to rest?* has similarities to the LOINC code

- *70815-6 | I need to rest during the day*

from the Functional Assessment of Chronic Illness Therapy (FACIT) Measurement System questionnaire, which is included in LOINC. Sometimes, we also identified multiple LOINC codes that partially matched the searched item from the EORTC questionnaire. For example, the question *Do you have any trouble taking a long walk?* could be matched to the LOINC code

- *61609-4 | Are you able to go for a walk of at least 15 minutes [PROMIS]* or
- *79006-3 | Are you able to walk more than a mile [PROMIS]*

Overall, we could not match 2 of 30 questions (7%) from the QLQ-C30 questionnaire and 9 of 23 (39%) from the QLQ-BR23 questionnaire. Similar to LOINC, we did not find a single question in the EORTC PROs that exactly matched the SNOMED CT terminology, but we did find at least 1 or more concepts that described the requested symptoms. For example, the QLQ-C30 question *Have you felt nauseated?* could be mapped to the SNOMED CT concept:

- *422587007 | Nausea (finding)|*

Overall, 29 of 30 questions (96%) of the QLQ-C30 and 18 of 23 (78%) of the QLQ-BR23 could be mapped to SNOMED CT terminologies. However, finding appropriate terminologies for response options in SNOMED CT and LOINC has been difficult. The EORTC Quality of Life Group explicitly states that the coupling of textual descriptions and numerical scores is paramount for using their PRO questionnaire concepts [25]. Thus, the simple use of numerical scales such as

- *1157272001 | Numeric grade on a scale of 1 to 4 (qualifier value)|* or

general adjectival modifiers such as

- *89292003 | Rare (qualifier value)|*

would not be sufficient to match the response options. Although the LOINC terminology includes response list codes that include textual descriptions and numerical scores (eg, *LL5215-0*), we could not find a list that correctly matched all scores and associated text.

Adverse events associated with CTCAE-scored drugs have been reported using terminologies such as the Medical Dictionary for Regulatory Activities (MedDRA) [37]. Thus, the CTCAE scoring incorporates certain elements of the MedDRA terminology [24]. In general, there is a mapping between MedDRA and SNOMED CT [37], which can also be used for our purposes. However, this mapping is not freely available. A manual test to find corresponding SNOMED CT identifiers for the first 50 CTCAE terms resulted in 6 missing terms. For the CTCAE response options, the parent concept and its child concepts exist in SNOMED CT:

- Parent concept: *273141005 | Severities (qualifier value)|*
- Child concept: *255604002 | Mild (qualifier value)|*
- Child concept: *6736007 | Moderate (severity modifier) (qualifier value)|*
- Child concept: *2448400 | Severe (severity modifier) (qualifier value)|*
- Child concept: *442452003 | Life threatening severity (qualifier value)|*

However, the last class *dead* of the CTCAE scores is not included in this severity scoring, and there is no direct relationship to adverse events or the meaning of each score for a specific symptom. We have mainly used the European version of SNOMED CT. Nevertheless, we would like to mention that the US version of SNOMED CT has fully equivalent concepts for the CTCAE scores:

- *46411000124101 | Common terminology criteria for adverse events grade 1 (finding)|*
- *446421000124109 | Common terminology criteria for adverse events grade 2 (finding)|*
- *446431000124107 | Common terminology criteria for adverse events grade 3 (finding)|*

However, even with this CTCAE-related scoring, definitions and symptom descriptions are needed for each CTCAE term.

In summary, it is generally challenging to map the exact wording of questions derived from PRO questionnaires to LOINC or SNOMED CT codes if the specific questionnaire is not included in the terminology. However, linking the response options when text is transformed into numeric scales is easier. However, questionnaires like those of the EORTC consortium declare that more is needed to represent numeric response options with the category descriptions [25]. When comparing the 2 ontologies, LOINC was better suited to map PRO questionnaires and their associated response options than SNOMED CT due to its text representation options.

### Questionnaire Licenses

Standardized questionnaires are often licensed, for example, from EORTC, and can only be used for patient surveys after obtaining permission. For commercial purposes, permission usually requires payment of a fee. This may prevent their questions and response options from being included in the SNOMED CT or LOINC terminologies. However, even if a licensed version is available, it should be noted that this does not mean that it is freely available for use. For example, LOINC indicates the copyright for terms for which copyright licenses must be considered. Thus, some licensed PRO questionnaires can be found in LOINC, such as the FACIT PRO questionnaire [38] with its 7 panels, including copyright and reference information. This is possible because the English version of FACIT is freely available [38] and can be included in terminology databases. Including at least 1 language version of the PROs in terminology databases would increase the international data exchange and its reusability.

In recent years, there has been a general movement toward “open access” and “open data,” and Creative Commons licenses as an extension of copyright law are seen as a way to protect the origin of data while making it available [39]. In this context, Ehrnsperger and Tietze [40] provide a broad collection of applied cases for patent pledges and open IP. These concepts may also be considered for licensed questionnaires in the future. Furthermore, initiatives such as the Medical Data-Models portal attempt to establish an open metadata repository to overcome the licensing problem [41].

Because standardized questionnaires, such as those of the EORTC Quality of Group, are designed to be culturally independent and have been tested for language comparability [26], linking the data processing to another language would not change the meaning of the data. Instead, the comparability of the data supports the original intent of the consortia that created each PRO. This reconciliation of different languages would require human effort, but it would be a first step in improving the reusability and comparability of PRO results across studies.

### Terminology Changes

Terminologies are not static. LOINC is currently updated twice a year. SNOMED CT International Edition will be updated monthly beginning in 2022. As a result, terms and concepts may become inactive, and definitions may change. Although the meaning of an identifier should be stable, we found LOINC codes assigned to FSNs with several different parts when comparing the 2.64 and 2.75 releases. For example, the LOINC code *18682-5* is mapped to the

- FSN *Ambulance claims attachment:Cmplx:Enctr:^Patient:Set* in the 2.64 release and to the
- FSN *Note:Find:Pt:Ambulance:Doc:Emergency medicine* in the 2.75 release.

So, in the 2.64 release, it was defined as 5 parts. In the current 2.75, it is defined as 6 parts. No part is identical to another.

### Time Resolution

Many PRO questionnaires have a general recall period of 7 days [27,42]. However, only some questions in the questionnaire are associated with this period. Some questions do not specify the exact recall period; others include it at the top of a panel but do not specify it for each question. Thus, a semantic matching of the PRO text may not correctly match a textual description that includes the time span, even though both may refer to the same thing. Because the recall time can dilute the remembered details [3], it is essential to include the period in the semantic terminology or at least include it in one of its compositional parts.

Another issue is the semantic representation of time. An example is the textual description of the general PRO recall time, which can be *past week* or *past seven days*. This semantic variation could be resolved using relationships that indicate the same meaning. Notably, some LOINC terms also include the time resolution. Thus, another way to address the issue of including time is to extend the questionnaire FHIR specification in combination with omitting the time range from some LOINC terms. LOINC extension requests could also help to enable full terminology binding of a questionnaire. As a workaround, SNOMED CT concepts could be used to describe the subject of questions semantically.

## Opportunities for Improvement

### Extension of Terminologies

The interoperability requirement aligns with the original idea of standardized PRO questionnaires that can be used in trials worldwide. However, considering their year of origin, it is evident that digitization aspects were not considered in their development, and most versions were designed in the classical pen-and-paper format. To overcome this limitation, the EORTC Quality of Life Group has developed guidelines for coupling available EORTC instruments with electronic devices [25]. A desirable next step would be to adapt the existing copyright of the PROs to allow at least 1 language version in the terminology databases, as is the case for the FACIT PRO [38].

If licensing issues are not the limiting problem, new codes can be proposed to extend the current databases. In the case of LOINC, the proposal may include new terms for the entire questionnaire, or if some content matches existing terms, they may be included in the submission form and sent to the Regenstrief Institute [17]. Like LOINC, SNOMED CT is also open to requests for additional concepts, coordinated through its members’ National Release Centers [16]. Because both terminologies, SNOMED CT and LOINC, are curated databases, it should be expected that not all extension requests will be accepted. In addition, there is a time lag between submitting an extension request and the final release. Special circumstances call for special measures. LOINC allows for advance release of terms for emergency situations (eg, pandemics or new technologies).

However, these prereleases will be reviewed for the next version release and may disappear again. For SNOMED CT, there are 2 other ways to provide additional data: extensions and reference sets [16]. Extensions can be created to support national, local, or organizational needs that may not have international relevance. Instead, reference sets can be made to customize and extend the content for specific needs. However, in addition to the fact that these extensions have limited availability, a disadvantage of these reference sets is that they must be maintained in the future to adapt to new international SNOMED CT releases, which requires more effort than requesting new identifiers.

### Combining LOINC and SNOMED CT

Manual mapping of PRO questions has shown a better match using question text from LOINC. If the requested terminology extension is denied or there is too much time between the request and the release, another way to handle questions without matching LOINC terms might be to find SNOMED CT concepts that at least describe the topic of the underlying question. This will not be as specific as a matching question, but it will at least semantically describe the topic of the question. SNOMED CT provides so-called postcoordinated expressions to logically combine multiple identifiers logically to represent a clinical idea at a higher level of detail [16]. A postcoordinated expression can increase the specificity of the topic description for a question. For example, the question *During the past week, have you vomited?* corresponding to the main concept *300359004 | finding of vomiting* is extended by the attribute *Temporal relationship* and the value *per week*, resulting in the following code:

- *300359004 |Finding of vomiting| : 260863009 |Temporal relationship| : 259038000 |per week|*

In the future, it will be possible to use postcoordinated expressions with concrete values such as integer and decimal numbers as attribute values, which are already included in the compositional grammar specification but are not yet available in the International Edition.

## Distinction From Other International Medical Terminologies

Here, we have focused on the general applicability of semantic terminologies for PRO questionnaires in the 2 comprehensive terminology systems for clinical settings, SNOMED CT and LOINC, from an international perspective.

However, we would also like to point out some other interesting terminologies that allow PRO mapping, such as the NCI Thesaurus or the MedDRA. While NCI Thesaurus mainly focuses on cancer and thus includes cross-links to CTCAE [43], MedDRA contains a lexicon for adverse events common in clinical trials [34].

A recent study attempted to map free text entries to MedDRA terms manually and found a match for 68% of the terms [34]. However, similar to our findings, the authors encountered the problem that the textual description of adverse events could fit several different terminology codes, but they were mutually exclusive.

Next, we would like to make a small excursion into the general use of these terminologies in the health care sector, where medical documentation is undergoing a paradigm shift. The structured documentation of health data should now facilitate the exchange of patient data and not only be used for statistical and administrative purposes such as billing [44]. This puts the patient at the center of attention [45]. The International Classification of Disease and Related Health Problems (ICD) code is a globally recognized system of medical terminology that provides uniform names for medical diagnoses [46]. Although the semantic terminologies such as SNOMED CT or LOINC are much more fine grained and could, therefore, replace established data collections such as the ICD in health care, they will certainly only be used in the near future in addition to the ICD codes, as the use of ICD codes is legally required [44]. This also requires additional SNOMED CT or LOINC codes that map to the currently accepted ICD code versions ICD-10 or, soon, ICD-11. Because the aim of this viewpoint was to find a reasonably accurate mapping of the PRO questionnaires analyzed, we did not map the PROs studied to the latest ICD-11 code. However, automatic mappings have been proposed for this task [47].

## Discussion

### Principal Findings

In general, using semantic terminologies is possible to improve the interoperability and reusability of PRO questionnaires and their associated response options. However, this task cannot be automated and requires human effort. In particular, the limited number of available languages limits the general idea of barrier-free interoperability. We have limited our mapping to the English versions of SNOMED CT and LOINC for these reasons. Although this is a limitation of our approach, we believe that this is the most comprehensive version [17,18] and that our results would be even lower for other languages. In addition, the exact mapping of the phrasing needs to be revised. Although the PRO questionnaires examined in SNOMED CT could only be mapped by symptom descriptions, some PRO questions with exact wording could be found in LOINC. Recognizing that we analyzed a limited number of PRO questionnaires, we would also like to highlight the positive development that some have already been entered into LOINC. One reason for not including PRO questionnaires is primarily due to licensing and copyright issues, which currently prohibit the inclusion of the PRO questions in terminologies. Including at least 1 language version of a licensed PRO is desirable. In this context, initiatives such as the MDM-Portal should be mentioned again, which try to provide open formats [41]. Although SNOMED CT offers a wide range of possible numeric response scales, none of the available scales combine numeric scores with textual descriptions required for PROs. Although such a coupling of scores and text is available in LOINC, none of the available scales perfectly matched the response options of the PROs analyzed. In addition, some questions within a questionnaire may have inverted scales, so care must be taken.

### Conclusions

In this study, we manually mapped the PRO questionnaires EORTC QLQ-C30 and QLQ-BR23 as well as the PROMIS-29 and a CTCAE-based questionnaire to the 2 widely used standardized terminologies in health care settings, SNOMED CT and LOINC. We showed that among the PRO questionnaires analyzed, only the PROMIS-29 is fully available in LOINC, whereas for the others, between 60.9% and 93.3% of PRO questions could be linked to the LOINC terminology. Although the PROMIS questionnaire is unavailable in SNOMED CT, the American version includes the CTCAE questions, and 78.3%-96.7% of the EORTC questions could be linked to SNOMED CT. Even more critical were our findings concerning the response options. Except for the PROMIS-29 response options in LOINC, which included a score coupled with displayed text, these responses were not available for other questionnaires in any of the terminologies.

It would be desirable to allow at least 1 language version per licensed questionnaire to be included in the terminologies or to use the open formats for future trials. Moreover, special attention should be paid to linking scores and displayed text in the terminologies, as strongly recommended by the original questionnaire settings.

On the basis of our analysis, we recommend LOINC for the future inclusion of additional PRO questionnaires due to its ability to include displayed text.

## Appendix

Multimedia Appendix 1 Mapping table for translation of questionnaires to SNOMED CT and LOINC.

## Abbreviations

CTCAE: Common Terminology Criteria for Adverse Events
EORTC: European Organization for Research and Treatment of Cancer
ePRO: electronic patient-reported outcome
FACIT: Functional Assessment of Chronic Illness Therapy
FHIR: Fast Healthcare Interoperability Resources
FSN: fully specified name
ICD: International Classification of Disease and Related Health Problems
LOINC: Logical Observation Identifiers, Names, and Codes
MedDRA: Medical Dictionary for Regulatory Activities
NCI: National Cancer Institute
NEMO: Nebenwirkungs-Management Onkologie (German)
PRO: patient-reported outcome
PRO-CTCAE: Patient-Reported Outcomes Version of the CTCAE
PROM: patient-reported outcome measure
PROMIS: Patient-Reported Outcomes Measurement Information System
QLQ-BR23: Quality of Life of Breast Cancer Patients Questionnaire
QLQ-BR45: Updated version of the Quality of Life of Breast Cancer Patients Questionnaire
QLQ-C30: Quality of Life of Cancer Patients Questionnaire
SNOMED CT: Systematized Nomenclature of Medicine Clinical Terms

## Data Availability

All data generated or analyzed during this study will be included in the published article (and its supplementary information files).
